# Effect of Different Freeze–Thaw Cycles and Fucoidan on Structural and Functional Properties of Lotus Seed Starch Gels: Insights from Structural Characterization and *In Vitro* Gastrointestinal Digestion

**DOI:** 10.3390/foods14183177

**Published:** 2025-09-12

**Authors:** Hongqiang Wu, Haoyu Wang, Yujia Ou, Baodong Zheng, Yi Zhang

**Affiliations:** 1 Engineering Research Center of Fujian-Taiwan Special Marine Food Processing and Nutrition, Ministry of Education, Fuzhou 350002, China; wuhongqiang1010@163.com (H.W.);; 2 College of Food Science, Fujian Agriculture and Forestry University, Fuzhou 350002, China; why2809@163.com; 3 School of Marine Biology, Xiamen Ocean Vocational College, Xiamen 361100, China

**Keywords:** Lotus seed starch, Fucoidan, Freeze–thaw, Structural characterization, *In vitro* digestibility

## Abstract

The influence of freeze–thaw (FT) cycling and fucoidan incorporation on the structural and digestive characteristics of lotus seed starch (LS) gels was systematically examined. Fucoidan–lotus seed starch (F-LS) gels were exposed to 0, 1, 3, and 5 FT cycles. Repeated FT treatments were found to disrupt the gel matrix and decrease thermal stability, whereas the addition of 1–2% fucoidan effectively alleviated these degradations. Crystallinity was significantly reduced from 37.62% to 26.38% (*p* < 0.05), indicating suppressed retrogradation. Thermal gravimetric and low-field NMR analyses revealed reinforced matrix cohesion. *In vitro* digestion assays demonstrated that fucoidan significantly retarded starch hydrolysis and promoted resistant starch (RS) formation. After five FT cycles, the RS content of 2% F-LS gels reached 29.03%, a 30.24% increase compared to the control. These findings suggest that fucoidan could serve as a natural and effective cryoprotectant and digestibility modulator in starch-based functional foods, offering both technological and nutritional benefits.

## 1. Introduction

Starch is extensively employed as a thickening and gelling agent in a wide range of ready-to-eat, pre-packaged, and frozen food products [1]. The increasing demand for convenient foods, driven by modern lifestyles, has accelerated the growth of the frozen and instant food sectors. Freezing is one of the most commonly adopted preservation techniques for starch-based products such as sauces, soups, frozen desserts, and gel-based snacks [2]. However, repeated freeze–thaw (FT) cycles during storage and transportation can markedly alter the microstructure and physicochemical properties of starch gels, often leading to undesirable changes in texture, phase separation (syneresis), and digestibility [3,4]. Typical freezing temperatures in freeze–thaw studies range from −20 °C to −80 °C, with −20 °C to −30 °C representing slow freezing that produces larger ice crystals and −80 °C representing rapid freezing that limits crystal growth. Thawing is generally performed at either low temperatures (4 °C) or moderate temperatures (25–30 °C), depending on whether structural preservation or rapid melting is prioritized [5].

Cryotropic gelation, induced by ice crystal formation and starch chain aggregation during freezing, results in sponge-like textures and phase separation upon thawing [6]. The migration of water and the retrogradation of amylopectin are exacerbated with increasing FT cycles, thereby compromising the quality and stability of starch-based food matrices [7]. In recent years, polysaccharide–starch composite gels have gained attention for their potential to improve freeze–thaw stability due to their superior gelling capacity and water-binding ability. Several studies have reported that the incorporation of functional oligosaccharides, such as xylooligosaccharides, galactooligosaccharides, and fructooligosaccharides, can enhance the stability of starch gels [8]. Nevertheless, further investigations are required to explore alternative bio-functional polysaccharides that may act as structural stabilizers under FT cycles.

Lotus seed starch (LS), a traditional starch source in Asian diets, is characterized by a high amylose content and favorable digestibility [9,10]. Despite these advantages, native LS gels show poor freeze–thaw stability and are prone to structural deterioration, limiting their broader application in frozen food systems [11]. Fucoidan, a sulfated polysaccharide extracted from brown seaweed, has attracted considerable interest due to its wide spectrum of biological activities, including antioxidant, anti-inflammatory, and prebiotic effects [12,13]. Given its strong hydrophilicity and ability to form hydrogen bonds, fucoidan is hypothesized to interact with starch molecules and potentially modulate gel structure, particularly under freezing conditions. However, the effects of fucoidan incorporation on the freeze–thaw behavior and digestibility of starch-based gels remain insufficiently understood. While the interactions between starch and non-starch polysaccharides under heating, gelatinization, and mechanical treatments have been explored, limited attention has been given to the structural and functional responses of fucoidan–lotus seed starch (F-LS) gels to repeated FT cycles.

In the present study, the impact of different freeze–thaw cycles and fucoidan concentrations on the structural integrity and digestive properties of F-LS composite gels was investigated. Microstructural and molecular-level changes were characterized using scanning electron microscopy (SEM), X-ray diffraction (XRD), thermogravimetric analysis (TGA), and low-field nuclear magnetic resonance (LF-NMR). Furthermore, a standardized *in vitro* digestion model was employed to assess starch digestibility. The findings provide mechanistic insights into the role of fucoidan as a structural stabilizer and digestion modulator in starch gels.

## 2. Materials and Methods

### 2.1. Materials

Lotus seeds were purchased from Green Acres (Fujian) Food Co., Ltd. (Sanming, China). LS was extracted following the previously established method of the research group [14]. Fucoidan (purity > 98%, Mn ≈ 18 kDa, L-Fucose 25.40%, Organic SO_4_^2−^ 25.70%) was purchased from Shanghai Aladdin Biochemical Technology Co., Ltd. (Shanghai, China). Information on purity, monosaccharide composition, and degree of sulfation was provided by the manufacturer (Supplementary File S1), while the molecular weight was obtained from previous reports [15]. Artificial gastric fluid (pH 3.0) and artificial intestinal fluid (pH 7.0) were purchased from Chuangfeng Automation Technology Co., Ltd. (Dongguan, China). 3,5-Dinitrosalicylic acid (DNS) reagent was purchased from Coolaber Science & Technology Co., Ltd. (Beijing, China). The starch content assay kit was obtained from Solarbio Science & Technology Co., Ltd. (Beijing, China).

### 2.2. Sample Preparation

Fucoidan was dissolved in distilled water at concentrations of 0%, 1%, and 2% (*w*/*w*, relative to the dry weight of lotus seed starch), and subsequently mixed with 6% lotus seed starch (*w*/*w*, based on the total sample weight). The addition levels of 1–2% were chosen with reference to previous literature [16] on polysaccharide–starch interactions and modified. The mixtures were stirred thoroughly at 95 °C for 30 min to ensure complete gelatinization. Subsequently, the samples were subjected to freeze–thaw cycles by freezing at −20 °C for 22 h and thawing at 30 °C in a water bath for 2 h, following commonly adopted conditions reported in previous studies [5]. This process was repeated for 1, 3, and 5 cycles. After treatment, the samples were freeze-dried, ground into powders, and stored in sealed containers for subsequent analyses.

The samples were named according to the number of FT cycles and fucoidan concentration. Specifically, samples without FT treatment were designated as 0FT-0%F-LS, 0FT-1%F-LS, and 0FT-2%F-LS; samples subjected to one cycle were named 1FT-0%F-LS, 1FT-1%F-LS, and 1FT-2%F-LS; samples subjected to three cycles were labeled 3FT-0%F-LS, 3FT-1%F-LS, and 3FT-2%F-LS; samples subjected to five cycles were labeled 5FT-0%F-LS, 5FT-1%F-LS, and 5FT-2%F-LS.

### 2.3. Structural Characterization of the F-LS Gel Following Multiple FT Cycles

#### 2.3.1. Scanning Electron Microscopy (SEM)

The microstructure of the samples was observed using a scanning electron microscope (Hitachi Regulus 8230, Hitachi, Tokyo, Japan) at a magnification of 5000×. Prior to observation, the samples were sputter-coated with a thin layer of gold to enhance conductivity.

#### 2.3.2. X-Ray Diffraction (XRD)

X-ray diffraction analysis was conducted using an Ultima IV diffractometer (Rigaku SmartLab SE, Rigaku, Japan). The measurements were performed using Cu-Kα radiation (λ = 1.54056 Å) over a 2θ range of 5–60°, with a scanning rate of 5°/min. The relative crystallinity of the samples was analyzed based on the obtained diffraction patterns [17].

(1) $$\mathrm{Relative}\;\mathrm{crystallinity}\;(\mathrm{RC})=\frac{A_{c\;}}{A_{a\;}+A_{c\;}}\;\times \;100\%$$

where A_c_ is the crystalline area and A_a_ is the amorphous area observed using XRD in PeakFit software 4.12 (SeaSolve SoftwareInc., Framingham, MA, USA).

#### 2.3.3. Thermogravimetric Analysis (TGA)

Thermal stability of the F-LS gels was evaluated using a thermogravimetric analyzer (TGA 8000, PerkinElmer, Shanghai, China). Approximately 5 mg of freeze-dried sample was placed in an alumina crucible and heated from 30 °C to 600 °C at a constant rate of 10 °C/min under a nitrogen atmosphere (flow rate: 20 mL/min) to prevent oxidative degradation [18]. The weight loss and thermal decomposition behavior were recorded continuously, and TGA and derivative thermogravimetric (DTG) curves were used to assess changes in thermal stability associated with different fucoidan concentrations and freeze–thaw cycles.

#### 2.3.4. Low-Field Nuclear Magnetic Resonance (LF-NMR)

Water distribution in the F-LS gels was analyzed using an LF-NMR analyzer (NMI20-060H-I, Niumag Co., Ltd., Shanghai, China) operating at a proton resonance frequency of 20 MHz. Approximately gel sample was placed in a 60 mm diameter cylindrical sample tube for measurement. The transverse relaxation time (T_2_) was determined using the Carr-Purcell-Meiboom-Gill (CPMG) pulse sequence, with a repetition time (TR) of 4000 ms, an echo time (TE) of 0.1 ms, and 16 scans per measurement.

The relaxation decay curves were fitted using a multi-exponential inversion algorithm provided by the Niumag NMR analysis software (Ver4.0). The resulting T_2_ components were interpreted to differentiate between bound water, immobilized water, and free water fractions within the gel matrix [19]. Changes in water distribution were used to assess the effects of freeze–thaw cycles and fucoidan incorporation on the internal water-holding structure of the gels.

### 2.4. In Vitro Gastrointestinal Digestion

The *in vitro* digestibility of F-LS gels was assessed using a two-stage simulated gastrointestinal digestion model adapted from established protocols [20,21]. Approximately 1 g of freeze-dried gel sample was dispersed in 1 mL of distilled water and thoroughly mixed. To simulate gastric digestion, 2 mL of simulated gastric fluid (pH 3.0), containing pepsin was added, corresponding to a final enzymatic activity of 2000 U per mL (equivalent to ~2000 U per g of sample) to the mixture. The mixture was incubated in a shaking water bath at 37 °C for 2 h with continuous agitation (e.g., 160 rpm). Subsequently, 4 mL of simulated intestinal fluid containing trypsin (100 U/mL, ~400 U/g sample) and amyloglucosidase (21 U/mL, ~84 U/g sample) was added. The mixture was briefly stirred for 5 s and the pH was adjusted to 7.0 using 0.1 M NaOH to mimic intestinal conditions. The digestion was continued at 37 °C for 3 h under constant agitation.

Aliquots of 0.2 mL were collected from the digestion supernatant at 0, 20, 30, 60, 90, 120, 150, and 180 min during the intestinal phase. Anhydrous ethanol was immediately added to each sample to inactivate the enzyme, and then centrifuged at 8000 rpm for 10 min. The aliquots of supernatant were used to determine the hydrolyzed glucose content using the 3,5-dinitrosalicylic acid method.

The hydrolysis rate was calculated according to the equations proposed by Englyst et al. [22]. The hydrolysis curves obtained from the intestinal phase were further fitted to a first-order kinetic model to enable direct comparison of digestion rates among treatments. The model was expressed as:

(2) $$\mathrm{Hydrolysis}\;\mathrm{rate}\;(\%)\;=\;\frac{G_{t\;}\;\times \;0.9}{\mathrm{TS}}\;\times \;100\%$$

where G_t_ is the amount of hydrolyzed glucose at time t. TS is the weight of the starch sample.

(3) $$C(t)=C_{0}+(C_{\infty }-C_{0})\;(1{-e}^{-kt})$$

where C(t) is the amount of starch hydrolyzed at time t, C_0_ is the initial hydrolysis at t = 0, C_∞_ is the equilibrium hydrolysis at the endpoint, and k is the first-order rate constant.

In addition, the contents of rapidly digestible starch (RDS), slowly digestible starch (SDS), and resistant starch (RS) were calculated according to the following equations [23]:

(4) $$\mathrm{RDS}\;(\%)\;=\;\frac{{(G}_{20\min\;}-{\;\;G}_{0\min\;})\;\times \;0.9}{\mathrm{TS}}\;\times \;100\%$$

(5) $$\mathrm{SDS}\;(\%)=\frac{{(G}_{120\min\;}-{\;\;G}_{20\min\;})\;\times \;0.9}{\mathrm{TS}}\;\times \;100\%$$

(6) RS (%)=(1−RDS−SDS) × 100%

where G_0min_, G_20min_ and G_120min_ refer to the amount of glucose released within 0, 20, and 120 min, respectively. TS is the weight of the starch sample.

### 2.5. Statistical Analysis

The experimental data were processed and the figures were generated using Origin 2022 software. The significance analysis was conducted using one-way analysis of variance (ANOVA) with Duncan’s test in SPSS 27.0 software. The data are presented as Mean ± SE (*n* = 3).

## 3. Results and Discussion

### 3.1. Microstructural Changes (SEM) of F-LS Gels

**Figure 1:**
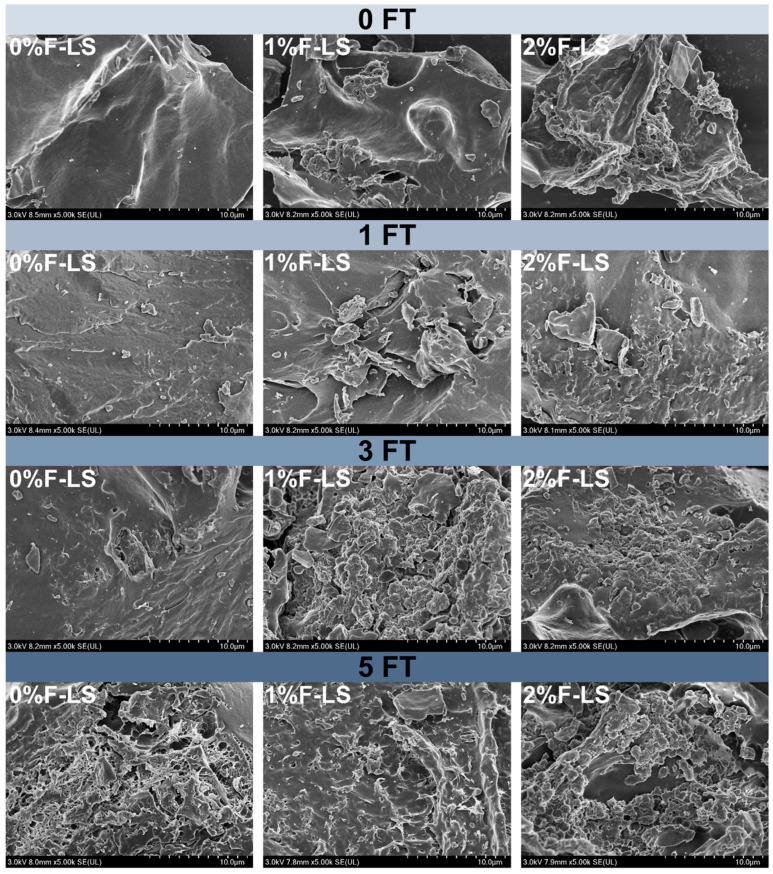
Microstructures of LS gels subjected to different FT cycles and fucoidan incorporation as observed by SEM.

The microstructural alterations of F-LS composite gels under different FT cycles were investigated using SEM, as shown in Figure 1. The control sample without fucoidan (0FT-0%F-LS) exhibited a dense and continuous matrix, characteristic of a native LS gel network. As the number of FT cycles increased, this structure progressively deteriorated. After one (1FT-0%F-LS) and three (3FT-0%F-LS) cycles, surface roughness and microfissures were observed, indicating the initiation of network disruption. Following five cycles (5FT-0%F-LS), pronounced structural collapse and fragmentation were evident. These changes were attributed to ice crystal formation and water migration during the FT process, which collectively induced internal mechanical stress and phase separation within the gel [24].

In fucoidan-containing gels, the extent of FT-induced damage appeared to be moderately alleviated in a concentration-dependent manner. LS Gels containing 1% fucoidan (e.g., 1FT-1%F-LS and 3FT-1%F-LS) maintained a slightly more cohesive morphology. Samples containing 2% fucoidan preserved a relatively compact and interconnected structure even after five cycles (5FT-2%F-LS). This stabilization was likely attributed to hydrogen bonding and electrostatic interactions between fucoidan and starch molecules, which reinforced the network and reduced susceptibility to ice-induced damage [12]. The occurrence of lamellar or stacked domains in fucoidan-containing gels further suggested a rearrangement of polysaccharide–starch regions during FT cycling, contributing to resistance against gel structure deterioration. These observations were broadly consistent with earlier studies reporting that hydrocolloids such as xanthan gum, konjac glucomannan, and alginate helped to mitigate microstructural deterioration of starch gels by reducing cavity formation and maintaining matrix integrity [25,26]. In summary, the incorporation of fucoidan moderately alleviated the microstructural deterioration of LS gels and reduced gel disintegration, thereby enhancing their tolerance to FT cycles to a certain extent.

### 3.2. Crystalline Structure (XRD) of F-LS Gels

**Figure 2:**
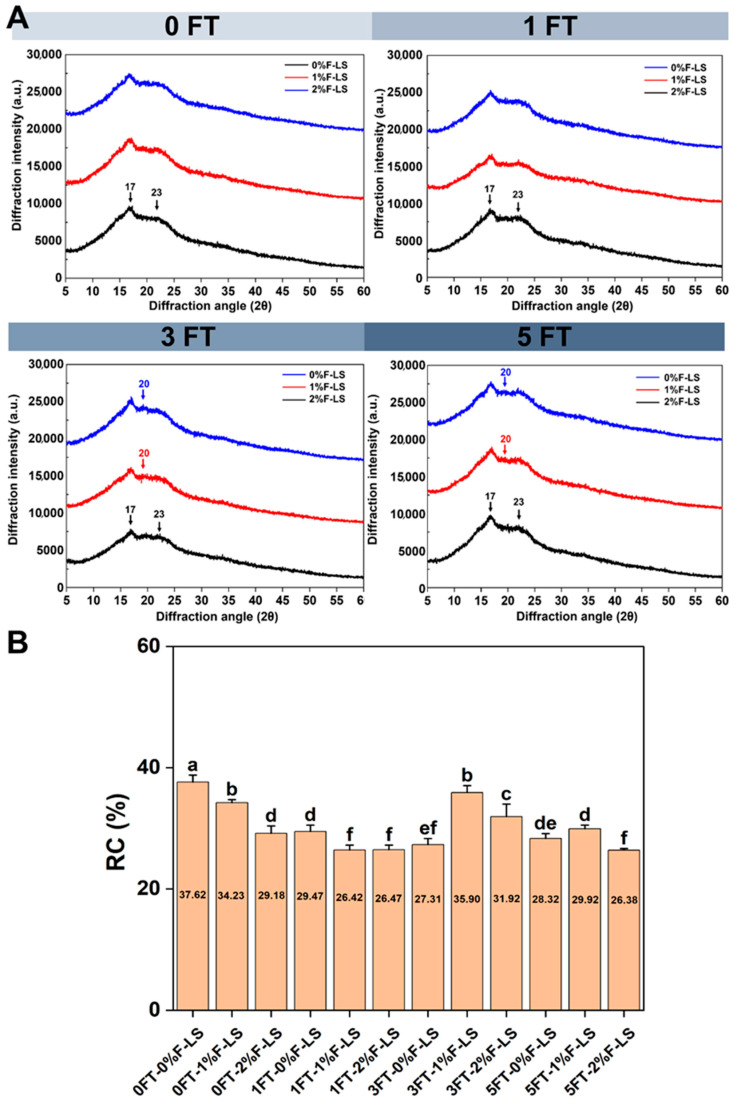
Crystalline structures of LS gels after various FT cycles and fucoidan incorporation as determined by XRD. (**A**) XRD patterns; (**B**) RC values. Different lowercase letters above the bars indicate significant differences among groups (*p* < 0.05).

The crystalline characteristics of F-LS gels subjected to varying FT cycles were analyzed using XRD, as depicted in Figure 2A. Native LS exhibited a typical C-type crystallinity, with diffraction peaks located at approximately 15°, 17°, 18°, and 23° (2θ) [27]. Upon gelatinization and subsequent cooling, a transition from C-type to B-type crystallinity occurred [28], as evidenced by the emergence of strong peaks around 17° and 23° in the 0FT-0%F-LS sample. This transformation was consistent with previous findings, confirming that thermal-cooling treatments facilitate B-type reassembly in LS gels. The incorporation of fucoidan at 1% and 2% did not alter the B-type crystalline pattern at 0 FT. However, after three and five FT cycles, a new, albeit weak, peak appeared near 20° (2θ), particularly in the 1% and 2% F-LS samples. This suggested the potential formation of V-type single helices [14], which may have resulted from specific interactions between fucoidan and amylose chains. Such interactions likely interfered with the reassociation of native starch chains, thereby slightly impeding retrogradation.

As shown in Figure 2B, the RC values of F-LS gels during FT cycles were observed. The control group (0FT-0% F-LS) exhibited the highest RC value (37.62, *p* < 0.05), indicating the high retrogradation tendency of LS. The RC values of LS gels without fucoidan decreased gradually with increasing FT cycles, showing a reduction of approximately 9.30% from 0 to 5 FT cycles, which suggested structural disintegration of the gel [29]. This result was consistent with SEM observations, confirming that the gel structure was progressively disrupted by FT cycling. Notably, after 3 FT cycles, the addition of fucoidan moderately mitigated the decline in crystallinity. This trend implied that fucoidan might interact with amylose to form transient single-helix structures, thereby reducing the structural damage induced by FT. The lowest RC value (26.38, *p* < 0.05) was observed in samples with 2% fucoidan after 5 FT cycles, indicating an inhibitory effect of fucoidan on the recrystallization of LS. This overall reduction in crystallinity could also be attributed to hydration-induced swelling and the steric or associative effects of hydrocolloids during gel formation, which interfered with starch retrogradation [30]. Such disruption has been regarded as advantageous for food applications, as it enhances textural stability and reduces syneresis. These findings were consistent with earlier reports showing that hydrocolloids suppressed retrogradation by hindering amylopectin recrystallization [31].

These results indicated that fucoidan incorporation interfered with the molecular alignment and recrystallization of starch chains during retrogradation. The steric hindrance and electrostatic repulsion associated with fucoidan likely impeded the close packing of amylopectin chains [32], thereby delaying, rather than completely preventing, structural deterioration of LS gels during repeated FT cycles.

### 3.3. Thermal Properties (TGA) of F-LS Gels

**Figure 3:**
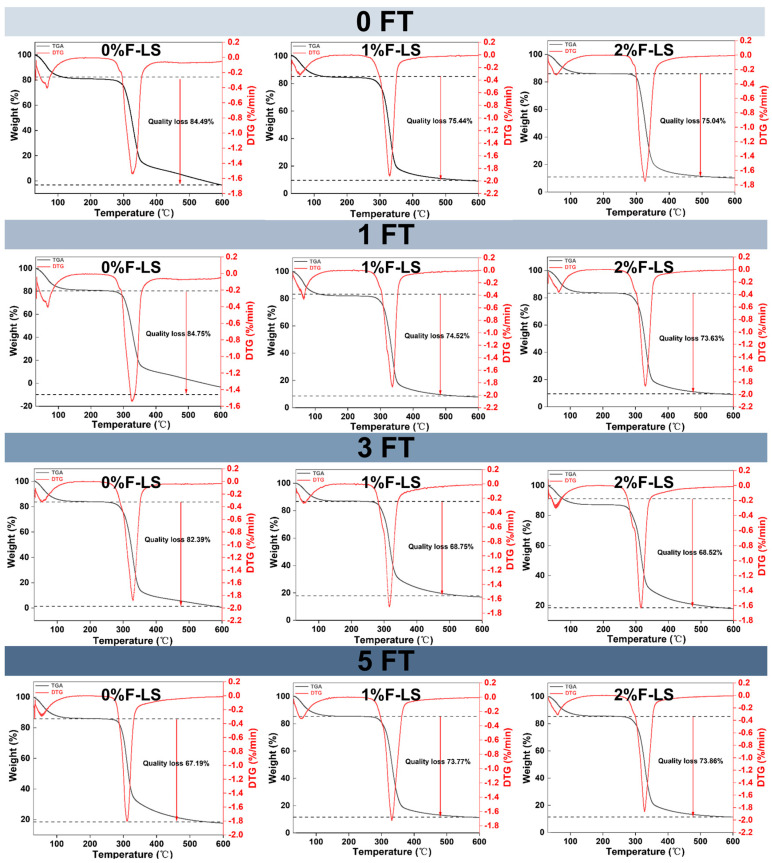
Thermal properties of LS gels subjected to different FT cycles and fucoidan incorporation as assessed by TGA.

The thermal stability of F-LS gels under different FT cycles was evaluated by TGA, and the corresponding weight loss curves are shown in Figure 3. All samples exhibited a two-step decomposition pattern. The first stage, occurring below 100 °C, was attributed to the evaporation of free and physically bound water [33]. The second, major weight loss stage was observed between 300 °C and 600 °C and was associated with the thermal degradation of starch and fucoidan macromolecules [34,35], including the cleavage of glycosidic bonds and depolymerization of the polysaccharide backbone.

At the second stage, the control group (0%F-LS) at 0 FT showed the highest total mass loss (84.49%), indicating poor thermal stability. In contrast, fucoidan alone exhibited greater thermal resistance, with a mass loss of only 45.79% at this stage (Supplementary File S2). When 1% and 2% fucoidan were incorporated, the total mass losses decreased to 75.44% and 75.04%, respectively. This reduction suggested that fucoidan contributed to the formation of a denser and more thermally stable gel matrix, likely through hydrogen bonding and molecular entanglement that restricted chain scission and promoted the generation of thermally stable residues [36]. These thermal changes were consistent with SEM observations of a more coherent network structure and with XRD results, which indicated a slight reduction in retrogradation. Comparable improvements in thermal stability have also been reported in starch–hydrocolloid systems, such as the incorporation of *Tremella fuciformis* polysaccharides, which enhanced the thermal resistance of *Castanea henryi* starch gels [37]. A similar trend was observed after 1FT and 3FT. In particular, after three cycles, the 1%F-LS and 2%F-LS gels retained relatively stable thermal properties, with the lowest mass losses of 68.75% and 68.52%, respectively. This indicated that fucoidan was able to buffer, at least partially, the detrimental effects of repeated freezing and thawing, as also reported previously [38].

After five FT cycles, the 0%F-LS gels exhibited the lowest apparent thermal stability, with mass loss decreasing to 67.19%. However, this did not reflect improved stability but rather severe network degradation, starch reassociation, and the accumulation of heat-resistant but structurally disordered residues. Such residues likely impeded complete thermal decomposition and resulted in an apparent reduction in weight loss. In contrast, the 1%F-LS and 2%F-LS gels maintained higher structural integrity, with mass losses of 73.77% and 73.86%, respectively. These results highlighted the protective effect of fucoidan, which helped preserve the thermal properties of LS gels throughout all FT cycles. This effect became more evident after multiple FT cycles (3FT and 5FT), where fucoidan-containing gels exhibited more consistent degradation patterns and better thermal resistance. Collectively, these findings, together with SEM and XRD observations, indicated that fucoidan mitigated FT-induced structural deterioration by stabilizing the gel network at both the microstructural and molecular levels.

### 3.4. Water Distribution (LF-NMR) of F-LS Gels

**Figure 4:**
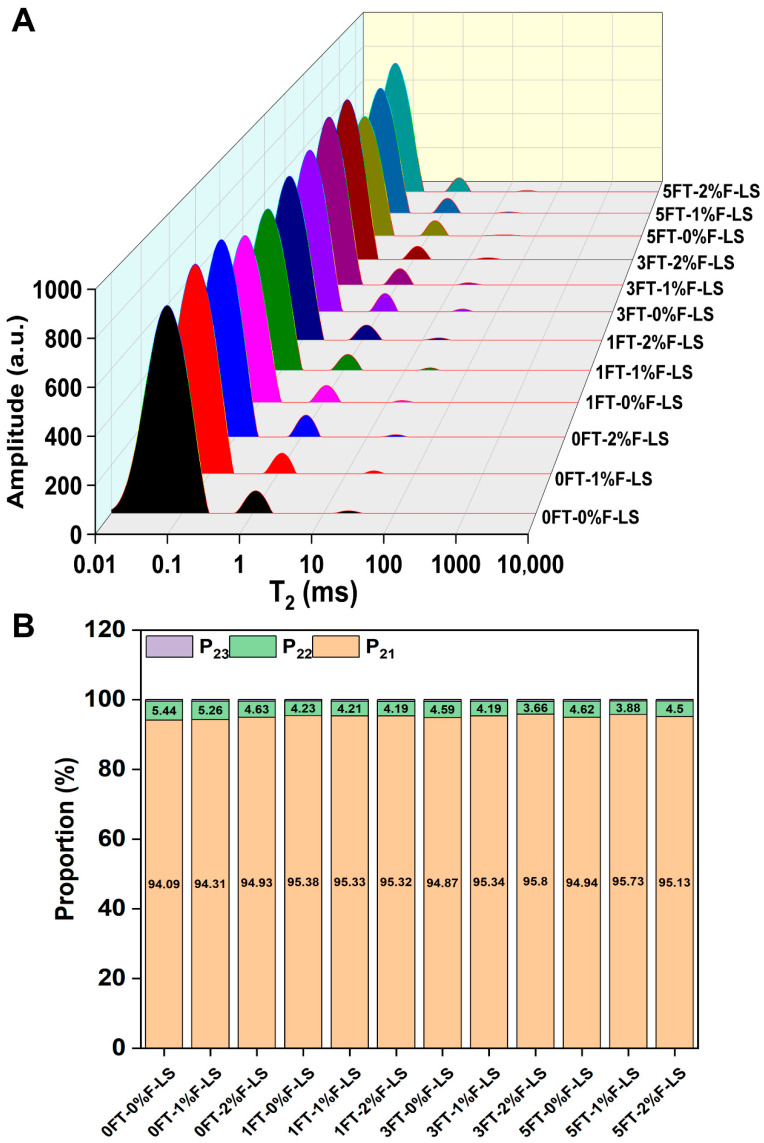
Water distribution of freeze-dried LS gels under different FT cycles and fucoidan incorporation based on LF-NMR analysis. (**A**) LF-NMR spectra; (**B**) Proportions of P_21_, P_22_, and P_23_.

Figure 4A displays the LF-NMR profiles of freeze-dried F-LS gels after various FT cycles. Due to lyophilization, the majority of free water (T_23_) was removed, resulting in negligible T_23_ peak intensities across all samples. Therefore, the analysis focused on bound water (T_21_) and immobilized water (T_22_), which reflected the hydration characteristics and the degree of molecular entanglement or confinement within the gel matrix [33].

As illustrated in Figure 4B, in LS gels without fucoidan, the proportion of T_22_ was initially relatively high, representing water entrapped within the gel network. However, with increasing FT cycles, the proportion of T_22_ gradually decreased, indicating that the gel network progressively lost its ability to retain immobilized water. This shift suggested that repeated freezing and thawing promoted water migration and exudation, leading to microstructural collapse and an increased risk of phase separation. In contrast, fucoidan-containing gels exhibited a different distribution pattern. Owing to the presence of abundant sulfate and hydroxyl groups, fucoidan displayed strong hydrophilicity and facilitated the retention of water molecules tightly associated with the macromolecular network [39]. Consequently, the relative proportion of T_21_ was slightly higher in the composite gels. More importantly, after five FT cycles, the relative proportion of T_21_ increased slightly in the 2%F-LS gels, while the T_22_ fraction was maintained. These results indicated that fucoidan contributed to the stabilization of the LS gel network and had the potential to improve its ability to retain immobilized water. Similar effects have also been observed for other hydrocolloids, such as deacetylated konjac glucomannan, which enhanced water immobilization and reduced syneresis in starch gels [40]. Overall, fucoidan incorporation slightly increased bound water and maintained immobilized water, thereby reinforcing their interactions and contributing to a more compact and stable polymer network during FT cycling.

### 3.5. In Vitro Digestibility of F-LS Gels

**Figure 5:**
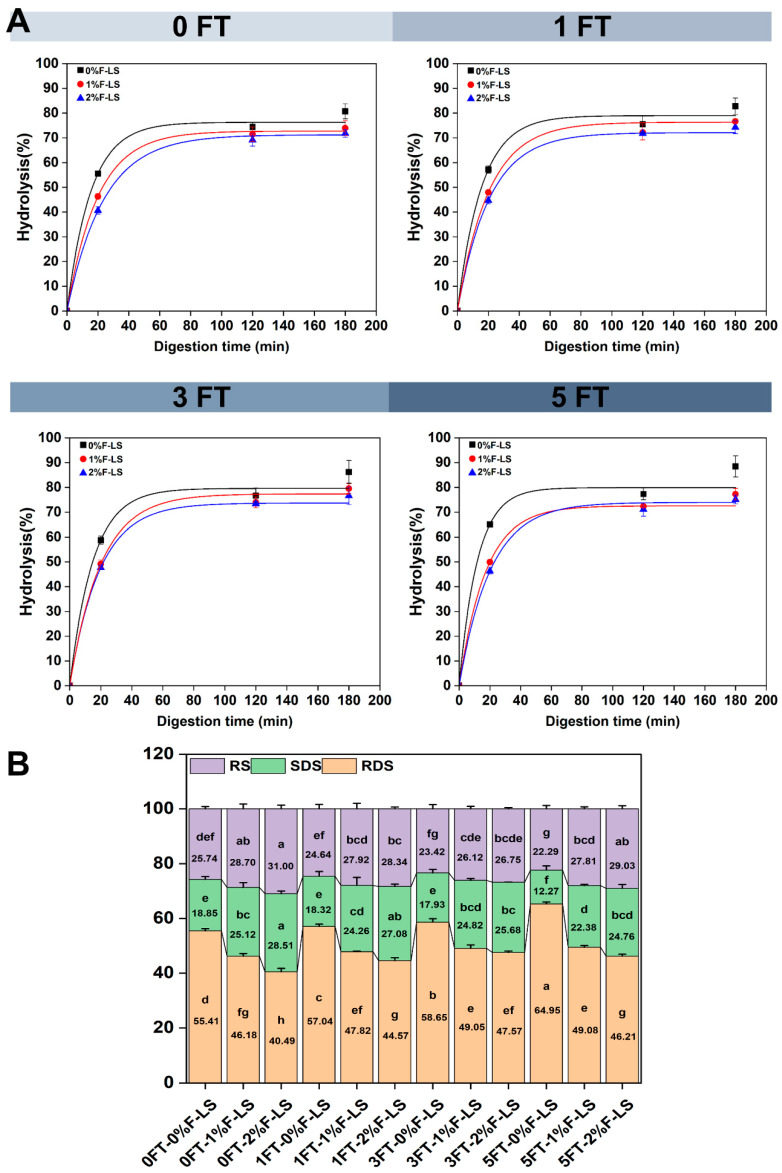
*In vitro* starch hydrolysis profiles of LS gels following various FT cycles and fucoidan incorporation. (**A**) Fitted hydrolysis curves; (**B**) Contents of RDS, SDS, and RS. Different lowercase letters above the bars indicate significant differences among groups (*p* < 0.05).

Figure 5A illustrates the fitted *in vitro* starch hydrolysis curves of F-LS gels under different FT cycles, while Figure 5B presents the corresponding RDS, SDS, and RS contents. The digestibility of LS was significantly influenced by both the number of FT cycles and the level of fucoidan incorporation. At 0FT, the control gel (0%F-LS) exhibited the highest hydrolysis rate, reaching 80.75% after 180 min, accompanied by a significant increase in RDS content (*p* < 0.05). This rapid digestion was attributed to the unstable structure of the LS gel. In contrast, the incorporation of 1% and 2% fucoidan significantly reduced the digestibility of LS gels, with final hydrolysis rates decreasing to 73.95% and 71.78%, respectively. Meanwhile, the RS content increased to 28.51% and 31.00%. These effects were consistent with the persistent and elevated T_21_ peaks observed in LF-NMR analysis, suggesting that fucoidan stabilized hydration shells and molecular entanglement, thereby restricting enzyme accessibility. Such hydrocolloid effects have generally been attributed to reduced enzyme accessibility to starch substrates, a protective mechanism commonly observed in polysaccharide–starch systems [41].

After one and three FT cycles, the digestibility of the control gels further increased, with hydrolysis rates reaching 82.73% and 86.22%, respectively. The RDS content also increased, reflecting cumulative structural damage, which was consistent with the structural collapse observed in SEM and the reduced thermal stability revealed by TGA. By contrast, the 1% and 2% F-LS gels maintained lower digestibility and higher SDS and RS contents, emphasizing that fucoidan continued to provide protection against enzymatic attack even under repeated FT cycling. This effect was mainly attributed to the steric barrier imposed by fucoidan, which limited the penetration of hydrolytic enzymes into starch granules. Similarly, calcium alginate has been reported to effectively reduce the digestibility of pea starch by creating a physical barrier that decreases the contact between amylolytic enzymes and starch molecules [42].

After five FT cycles, the differences became more pronounced. The 0%F-LS gels exhibited extensive degradation, with RDS reaching 64.95%, indicating severe network breakdown. In comparison, the 2% F-LS sample exhibited the slowest hydrolysis rate along with the highest SDS and RS contents. After five FT cycles, the RS content reached 29.03%, representing a 30.24% increase compared to the control group, which further demonstrated the protective effect of fucoidan under multiple FT cycles. Previous studies have also reported that fucoidan regulates starch digestibility by reinforcing the gel matrix, suppressing retrogradation, stabilizing water interactions, and reducing enzyme accessibility [43]. Collectively, these findings supported the potential application of fucoidan in the design of starch-based gels with enhanced resistance to digestion and possible benefits for glycemic control.

## 4. Conclusions

In this study, the effects of FT cycles and fucoidan addition on the structural and functional properties of LS gels were systematically investigated. The microstructure was found to be disrupted by repeated FT treatments, while the incorporation of fucoidan effectively stabilized the gel network. XRD and TGA results indicated that starch retrogradation and thermal degradation were suppressed by fucoidan. LF-NMR data revealed enhanced water binding. Importantly, *in vitro* digestion assays showed that fucoidan significantly reduced the starch hydrolysis rate and increased the RS content, even after multiple FT cycles. Collectively, these findings confirmed that fucoidan acted as a structural stabilizer and functional modulator in LS gels, improving their freeze–thaw stability and digestibility. Overall, fucoidan demonstrates promising potential as a natural additive for enhancing the performance of starch-based frozen foods. However, this study did not address its industrial scalability, potential interactions with other food ingredients, or sensory properties, which require further investigation.

## Data Availability

The original contributions presented in this study are included in the article/Supplementary Materials. Further inquiries can be directed to the corresponding author.

## Supplementary Materials

The following supporting information can be downloaded at: https://www.mdpi.com/article/10.3390/foods14183177/s1, Supplementary File S1: Certificate of analysis for Fucoidan; Supplementary File S2: Thermal properties of fucoidan as assessed by TGA.
