# Supplementary material for: Effect of Different Freeze–Thaw Cycles and Fucoidan on Structural and Functional Properties of Lotus Seed Starch Gels: Insights from Structural Characterization and In Vitro Gastrointestinal Digestion
Source: Foods. 2025 Sep 12;14(18):3177. doi: 10.3390/foods14183177 (PMC12470072; doi:10.3390/foods14183177)
Supplement: Supplementary file 1 [file foods-14-03177-s001.zip › foods-3805627-supplementary.pdf]

Sale@ aladdin-e . com

Shanghai Aladdin Biochemical Technology Co., LTD
809 Chu hua Branch Road, Fengxian District, Shanghai 201400, China

Certificate of Analysis

Date of Testing:	2025-04-17 10:18:37
Date of Next Testing:	2028-04-16 10:18:37
Product Name:	**Fucoidan**
CAS Number:	9072-19-9
Specifications & Purity:	≥98%
Lot #:	D2514340
SKU #	**F304947**
Version	**3**
**Analysis #:**	**488061**
Storage Temperature:	Store at 2-8°C

**Parameter**	**Limit Values**	**Result**
Loss on drying	0-10 (%)	8.19729 %
Organic SO_4^{2-}	20-100 (%)	25.70000 %
L-Fucose	20-100 (%)	25.40000 %
Purity(HPLC)	98-100 (%)	98.20000 %
Appearance (F304947)	White to Beige and Faint Brown to Brown Powder or crystal	White to Beige and Faint Brown to Brown Powder or crystal

John.Su

John Su
QA & QC Manager

Supplementary File 2

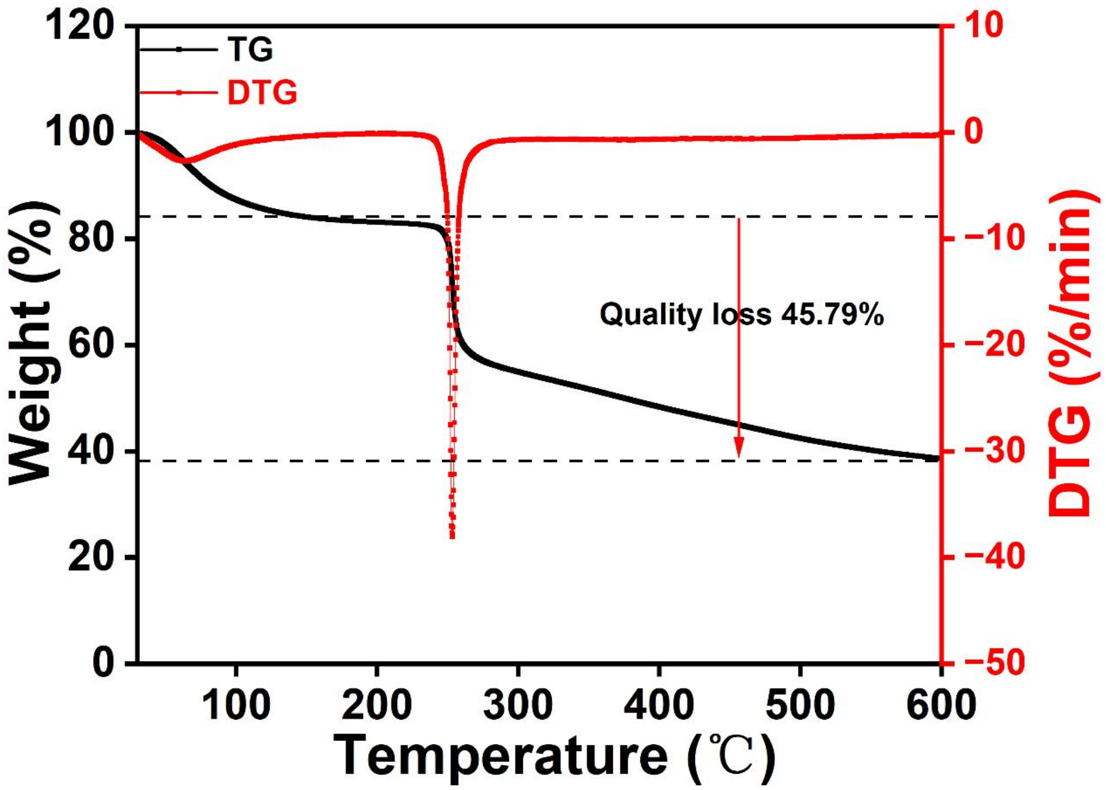

Supplementary File 2. Thermal properties of fucoidan as assessed by TGA.
